# Smell and taste of milk during tube feeding of preterm infants: neurodevelopmental follow-up of the randomized TASTE trial, study protocol

**DOI:** 10.1186/s13063-023-07224-0

**Published:** 2023-04-22

**Authors:** Friederike Beker, Ian P. Hughes, Sue Jacobs, Helen G. Liley, Samudragupta Bora, Gabrielle Simcock, Peter G. Davis

**Affiliations:** 1 Mater Research Institute, Faculty of Medicine, The University of Queensland, South Brisbane, Queensland Australia; 2 Neonatal Critical Care Unit, Mater Mothers’ Hospitals, South Brisbane, Queensland Australia; 3 Office of Research Governance and Development, Gold Coast University Hospital, Southport, Queensland Australia; 4 Neonatal Services and Newborn Research, The Royal Women’s Hospital, Melbourne, Victoria Australia; 5 Clinical Sciences Research, Murdoch Children’s Research Institute, Parkville, Victoria Australia; 6 Department of Obstetrics and Gynaecology, University of Melbourne, Melbourne, Victoria Australia; 7 Department of Pediatrics, University Hospitals Rainbow Babies & Children’s Hospital, Case Western Reserve University School of Medicine, Cleveland, Ohio USA; 8 School of Applied Psychology, Griffith University, Southport, Queensland Australia

**Keywords:** Bayley-4, Early nutritional learning, Follow-up, Neurodevelopment, Preterm

## Abstract

**Background:**

The Taste And Smell To Enhance nutrition (TASTE) trial investigated the effects of smell and taste of milk with tube feeding compared to routine care on the growth of preterm infants. There was no difference between groups in growth (weight, head circumference, length) *z*-scores at discharge from the hospital. Infants in the intervention group had higher head circumference and length *z*-scores at 36 weeks postmenstrual age, both secondary outcomes. The objective of this follow-up study is to assess 2-year neurodevelopmental and growth outcomes after exposure of preterm infants to the smell and taste of milk with tube feeding compared to routine care.

**Methods:**

This is a neurodevelopmental follow-up study of a two-center, placebo-controlled randomized trial. Infants born before 29 weeks postmenstrual age and/or with a birth weight of less than 1250 g were randomized to smell and taste of milk with each tube feed or routine care. The current follow-up assessed the 2-year neurodevelopmental and growth outcomes of participants of the TASTE trial discharged from the hospital (*n* = 334). The primary outcome is survival free of any major neurodevelopmental impairment comprising any moderate/severe cerebral palsy (Gross Motor Function Classification System score II–V), Bayley Scales of Infant and Toddler Development, Third/Fourth Edition (Bayley-III/Bayley-4) motor, cognitive, or language scores < -2SD, blindness, or deafness at 2 years of age. Other outcomes include death, breastfeeding within the first year, and respiratory support, oral feeding, and anthropometric parameters at 2 years of age. The Human Research Ethics Committees of Mater Misericordiae Limited and the Royal Women’s Hospital approved the TASTE trial including the neurodevelopmental follow-up described in this protocol.

**Discussion:**

For patients and their families, the neurodevelopmental outcomes of preterm infants are of utmost importance. Consequently, they should be investigated following any interventional study performed during the newborn period. Furthermore, improved weight gain and head growth in the hospital are associated with better long-term neurodevelopmental outcomes. Smelling and tasting of milk is an uncomplicated and cost-effective intervention that may improve the growth and neurodevelopmental outcomes of preterm infants. Potential limitations affecting this follow-up study, caused by the COVID-19 pandemic, are anticipated and discussed in this protocol.

**Trial registration:**

Name of the registry: Australian and New Zealand Clinical Trials Registry; Registration number: ACTRN12617000583347; Registration date: 26 April 2017.

## Background

Preterm birth is a strong predictor of poor health outcomes and is associated with long-term neurodevelopmental challenges [1–3]. Clinicians are continuously refining neonatal care to improve long-term outcomes for preterm infants. Optimizing nutrition is a focus as growth failure is a universal problem in this vulnerable population. Improved weight gain and head circumference during the initial hospital admission of preterm infants are associated with improved long-term neurodevelopmental outcomes, as long-term follow-up studies as late as early adulthood have demonstrated [4, 5].

Smell and taste sensations are primarily processed in the olfactory and gustatory cortex and then integrated with higher brain functions [6, 7]. The stimulation of those primary senses activates complex pathways and initiates food anticipation and cephalic phase responses that make up the orchestrated physiological preparation of the body for food intake [8–10]. “Early nutritional learning” describes interactions with food, including smells, tastes, and emotions around feeding that modify appetite and satiety development and the rate of weight gain [10–13]. It occurs during a time of brain development that is marked by rapid neuronal proliferation, myelination, and synaptogenesis and is known to generate the long-term basis for food preferences and eating behavior with effects seen even into adulthood [14–17]. Research into the long-term effects of early nutritional experiences is sparse but investigating the potential impact on cognitive outcomes has been suggested [18].

The Taste And Smell To Enhance nutrition (TASTE) trial evaluated the effects of smell and taste of milk with tube feeding versus routine care in preterm infants on weight at discharge [19]. A total of 396 infants born at less than 29 weeks postmenstrual age and/or with a birth weight of less than 1250 g were randomized to either smell and taste of milk with each tube feeding or routine care. It concluded that preterm infants with the provision of regular smell and taste of milk with a tube did not have higher growth (weight, length, head circumference) *z*-scores at discharge home from the hospital. In prespecified secondary analyses, we found that the intervention group had higher head circumference and length *z*-scores at 36 weeks postmenstrual age.

Smell and taste with tube feeding have no known side effects, require little extra time, and are easy to apply, with minimal costs. Given that late gestation and early infancy is a crucial time for brain development, nutritional learning experiences may be part of the more general neurodevelopmental journey of an infant [20–22].

With the additional observation in mind that infants in the TASTE trial’s intervention group have improved head circumference at 36 weeks postmenstrual age, a neurodevelopmental follow-up study of newborn infants exposed to oropharyngeal colostrum or smell and taste of milk, compared to routine care, is warranted. Moreover, for patients and their families, the neurodevelopmental outcomes of preterm infants are of utmost importance and should be investigated as part of any neonatal interventional study. The current follow-up study aims to assess long-term effects on neurodevelopment at 2 years corrected age (CA) of exposure to smell and taste of milk with tube feeding versus routine care in infants born at less than 29 weeks postmenstrual age and/or with a birth weight of less than 1250 g.

## Methods

### Study design

This is a follow-up study of a clinical, non-blinded, parallel-group randomized superiority trial (TASTE trial, ACTRN 12617000583347) [19]. The TASTE trial recruited participants between May 2017 and February 2020 at two Australian sites: the Mater Mothers’ Hospital in Brisbane, Queensland, and the Royal Women’s Hospital in Melbourne, Victoria. With parental consent, infants born at less than 29 weeks postmenstrual age or with a birth weight of less than 1250 g were randomized to receive the smell and taste of milk with tube feeding (intervention group) versus tube feeding alone (control group) until they were fully suck feeding or until discharged from hospital. A total of 396 infants were randomized: 196 were assigned to the intervention group, and 200 were assigned to the control group. No between-group differences were observed for the primary outcome, weight *z*-score at discharge [19]. However, in prespecified secondary analyses, head circumference and length *z*-scores were significantly higher in the intervention group at 36 weeks postmenstrual age.

Neurodevelopmental and growth outcomes were listed as secondary outcomes in the original TASTE trial protocol [23]. The current protocol for the 2-year follow-up study defines impairment classifications for outcomes and the statistical analysis as well as takes into account the anticipated impact of the COVID-19 pandemic, details not available in the original protocol. This follow-up protocol does not describe the original trial's randomization, intervention, or discontinuation or modification of the allocated intervention; those details can be found in the original TASTE trial protocol [23].

### Study setting

Participants who were enrolled in the TASTE trial as newborn infants and randomized to either smell and taste of milk with tube feeding or routine care had their neurodevelopmental outcome assessed at 2 years CA (defined as 2 years from the date the infant was expected to be 40 weeks gestation). There was no anticipated harm and no compensation for trial participation or provision for post-trial care. Data collection for this follow-up study was impacted by the COVID-19 pandemic, and therefore, the CONSORT and SPIRIT Extension for RCTs Revised in Extenuating Circumstances (CONSERVE 2021) is followed for the current protocol. The follow-up study was performed within the routine neonatal follow-up programs of the Mater Mothers’ Hospital in Brisbane and the Royal Women’s Hospital in Melbourne. Follow-up commenced in August 2019 and concluded in October 2022.

### Eligibility

Surviving infants who participated in the TASTE trial and were eligible for a routine clinical neurodevelopmental assessment qualified for inclusion in this follow-up study. In agreement with the Australian and New Zealand Neonatal Network recommendations, infants born at less than 28 weeks postmenstrual age and/or less than 1000 g birth weight are eligible for routine neurodevelopmental follow-up at 2 years CA [2]. No additional funding was available to ensure neurodevelopmental follow-up for TASTE trial participants who did not meet the Australian and New Zealand Neonatal Network criteria. There were no exclusion criteria.

### Assessment

At 2 years CA, participants underwent a standardized neurological assessment. Participants were assessed in a single session by a trained examiner, who was blinded to the intervention group allocation. Cerebral palsy was diagnosed using standard criteria, including gross motor impairment according to the Gross Motor Function Classification System (GMFCS) [24, 25]. Blindness was diagnosed when vision was worse than 6/60 in the better eye and deafness if amplification or cochlear implant were required. The Bayley Scales of Infant and Toddler Development, Third and Fourth Edition (Bayley-III/Bayley-4) assessed cognitive, language, and motor development [26].

### Outcomes

The primary outcome is survival free of moderate or severe neurodevelopmental impairment. The primary outcome will also be assessed within each stratifying variable: postmenstrual age at birth (< 27 or ≥ 27 weeks), sex, and study hospital. Moderate disability comprises moderate neurodevelopmental delay (Bayley-III/Bayley-4 cognitive, motor, and/or language composite scores < -2 to -3 SDs) or moderate cerebral palsy (walking at 2 years with the need for an assistive technology device, GMFCS level II or III). Severe disability comprises severe neurodevelopmental delay (Bayley-III/Bayley-4 cognitive, motor, and/or language composite scores < -3 SDs) or severe cerebral palsy (not walking at 2 years and not expected to walk, GMFCS level IV and V), blindness, or deafness. Participants for whom disability status cannot be determined (i.e., those with missing data for one or more primary outcome components and no disability on the non-missing components) will be excluded from the primary outcome analysis.

Secondary outcomes include:

1. Death before 2 years of age
2. Rate of mild, moderate, and severe disability. Mild disability comprises mild neurodevelopmental delay (Bayley-III/Bayley-4 cognitive, motor, and/or language composite scores < -1 to -2 SD) or mild cerebral palsy (walking at 2 years without limitations but signs of cerebral palsy, GMFCS I)
3. Mild, moderate, and severe cerebral palsy (GMFCS)
4. Mild, moderate, and severe neurodevelopmental delay (Bayley-III/Bayley-4)
5. Centiles of the Bayley-III/Bayley-4 cognitive, language, and motor composite scores
6. Head circumference, weight, length, and respective *z*-scores at 2 years of age
7. Partial or exclusive breastfeeding and/or presence of a nasogastric tube or percutaneous endoscopic gastrostomy feeding at 3, 6, and 12 months
8. Respiratory support at 2 years of age
9. Oral feeding at 2 years of age

### Sample size

The TASTE trial sample size of 330 infants had 90% power to detect a difference of 0.21 weight *z*-scores at discharge, considering multiples from one pregnancy as a cluster for a 2-sided alternative hypothesis using a generalized estimating equations model. In the TASTE trial, a total of 334 infants were discharged from the hospital and assessed for the primary outcome; however, only 270 of these were eligible for routine clinical follow-up at 2 years CA (< 28 weeks postmenstrual age and/or < 1000 g birth weight). Assuming a similar follow-up rate of 90% to that seen in cohorts at 2 years CA from the Australian state of Victoria, this follow-up study was thus expecting 243 participants. Assuming the same cluster size as for the original study, 1.09, and an intra-cluster correlation of 0.69 (based on that for weight *z*-score at discharge), we have more than 80% power to detect a difference of 13.7% (87.7% smell and taste group, 74% routine care group) at a significance level of 0.05 in the primary outcome of this follow-up study (survival free of moderate or severe neurodevelopmental impairment at 2 years CA) using a logistic generalized estimating equations analysis. A difference of 13.7% would, however, be considered larger than a minimum clinically significant effect but this sample size is not large enough to detect smaller differences reliably [27]. This trial's outcome data could potentially be used in meta-analyses of trials with similar interventions and outcomes, as well as informing the development of future studies.

### Recruitment

Clinical staff routinely cross-checked medical records for possible deaths before inviting a family to a routine follow-up appointment.

### Allocation

This is the follow-up study for the TASTE trial. The original TASTE trial was open-label, whereas the outcome assessors in the current follow-up study were blind to the intervention. Details regarding the trial conduct auditing, sequence generation, allocation concealment mechanisms, implementation, and discussion of the absence of blinding the intervention are available in the TASTE trial protocol and publication [19, 23].

### Data management

Parents specifically consented to data collection of the current follow-up when they consented to the original TASTE trial. This follow-up study did not involve the collection of biological specimens for storage. Data were sourced from clinical care team notes, medical records, and from parents directly. Each infant has been assigned a study number when recruited to the TASTE trial and data were collected under that study number. Individual participant identifiers apart from study numbers were removed when data were entered onto a paper case record form, then transferred by the data manager to an Excel spreadsheet and stored on a password-protected computer on the Mater Mothers’ Hospital’s computer network. Each data set will be checked by the principal investigator for plausibility and data range checks are applied in the database where appropriate. A data-sharing agreement has been established between the two participating hospitals, and all study group members have access to the final data set.

### Statistical methods

Statistical analysis will be performed by the authors Hughes and Beker with the assistance of other study group members. Data will be exported from an Excel spreadsheet to a statistical package for analysis (Stata 17; College Station, Texas, USA). Data will be analyzed on an intention-to-treat basis. All randomized infants will be included in the primary analysis unless consent has been withdrawn.

There will likely be some substantial missing data as this is a follow-up study. Initially, patterns of missingness of data will be assessed and this information used to determine if missingness is likely to be completely at random (MCAR; missingness is unrelated to values of any known or unknown variable), at random (MAR; missingness of a variable’s values is dependent on known variables other than itself), and not at random (MNAR; missingness depends on the value of the missing value). We plan to use multiple imputation (mi suite of commands in Stata) to impute missing data and jointly analyze the multiple imputed datasets. These methods assume a MAR mechanism of missingness. In practice, it is difficult to definitively show that a variable is MAR rather than MNAR. As such, sensitivity analyses will be performed to see how specific violations of this assumption affect the outcome.

The primary outcome, survival free of moderate or severe neurodevelopmental impairment, and other outcomes with categorical data will be analyzed using a logistic generalized estimating equations analysis (xtlogit Stata command) and subgroup analyses of the primary outcome will be undertaken within each stratum of the stratifying variables (sex, postmenstrual age at birth, birth hospital). Univariate and where appropriate multivariate generalized estimating equations analyses will be used for continuous secondary outcome measures (xtgee command). All outcomes will be assessed against an alternative hypothesis of superiority.

### Dissemination of results

The results of the trial will be published in a peer-reviewed journal and will be presented at national and international conferences. Authorship will be determined in line with the International Committee of Medical Journal Editors guidelines.

## Discussion

This neurodevelopmental follow-up study was affected by the COVID-19 pandemic. Melbourne was in prolonged lockdown while Brisbane was affected by extensive travel restrictions and several shorter regional lockdowns. Neurodevelopmental follow-up clinics were never closed, but some study families were unable to travel the required distances, did not attend hospital appointments to avoid potential exposure to COVID-19, or were otherwise affected by the COVID-19 pandemic [28]. These extenuating circumstances impacted this 2-year follow-up study.

During the COVID-19 pandemic, some clinical trials were paused to manage the public health crisis. This follow-up study was embedded into the routine neonatal follow-up program of the respective hospital. Follow-up assessments were not interrupted by public health measures at the Mater Mothers’ Hospital in Brisbane, but at the Royal Women’s Hospital in Melbourne, several prolonged lockdowns made it impossible for trial participants to attend outpatient appointments, or outpatient follow-up was canceled. Therefore, this follow-up study will have missing data, leading to a reduction of statistical power to detect a clinically important difference between groups.

Enrollment of a smaller proportion than planned may result in bias, thus compromising the generalizability of the results. Loss of power will be evaluated and discussed in the final report of the follow-up study. In addition, a table comparing study participants with complete data and those without complete data, stratified by the intervention, will be presented. Regardless of the likely presence of attrition bias, the TASTE trial presents a unique opportunity to obtain longer-term outcomes and may inform the design of future trials.

If missingness of follow-up data is plausibly viewed as *missing completely at random*, meaning that the missingness is independent of any measured or unmeasured participant characteristic, attrition bias is unlikely. If missingness is likely *missing at random*, an appropriate statistical analysis method to control for potential bias, such as inverse-probability weighting or multiple imputations, will be applied. If the missingness is plausibly not missing at random, a definitive bias adjustment may not be possible, and researchers will consider using appropriate sensitivity analysis methods [29].

## Trial status

The trial sponsor, Mater Misericordiae Limited, provided funding, a human research ethics committee, and governance services. The trial was coordinated by the principal investigator, who was assisted by research nurses from the neonatal intensive care unit. The recruitment of participants and the intervention were completed as part of the original TASTE trial and are not included in this follow-up protocol. Meetings with the research nurses were not scheduled regularly but instead took place as needed. This follow-up phase of the trial did not have a Trial Steering Committee or a Stakeholder and Public Involvement Group. The original TASTE trial did not have a Trial Steering Committee because it was deemed low risk. The Human Research Ethics Committees of Mater Misericordiae Limited and Royal Women’s Hospital approved the study protocol of the original TASTE trial (version 3, 8 May 2017), trial reference number HREC/16/MHS/112 and trial reference number 17/21, respectively, and review the trial progress and conduct yearly. This original trial protocol included the collection of 2-year neurodevelopmental follow-up data. Both hospitals also granted governance approval. The primary outcome of the TASTE trial was assessed at the time of discharge from the hospital. The outcome of this TASTE trial follow-up study was assessed at the routine neurodevelopmental follow-up clinic appointment occurring at the respective hospital. Follow-up commenced in August 2019 and concluded in October 2022. The timeline diagram is the same as available in the original TASTE trial protocol [23].

## Data Availability

Data from the current follow-up study will not be eligible for public data sharing due to the constraints of consent from the original trial. The Governance Office of Mater Research can provide further information: agreements@mater.uq.edu.au.
